# Supplementary material for: Why chimpanzees carry dead infants: an empirical assessment of existing hypotheses
Source: R Soc Open Sci. 2020 Jul 1;7(7):200931. doi: 10.1098/rsos.200931 (PMC7428235; doi:10.1098/rsos.200931)
Supplement: Supplementary Methods [file rsos200931supp1.docx]

**Electronic Supplementary Materials**

METHODS

*Study communities and data sources*

Historically, the Kasekela community has ranged in size from 38-62 individuals, with age-sex classes ranging from 7-14 mature males, 12-25 mature females, and 13-29 immatures (< 12 years of age). The Mitumba community has ranged in size from 19-29 individuals, with age-sex classes ranging from 2-5 mature males, 5-10 mature females, and 10-15 immatures.

Standardized focal behavioral data has been collected on a near-daily basis since the early 1970s. Researchers attempt to follow the majority of mature chimpanzees in each community at least once per month. During these follows, a team of two researchers record group composition and location every 15 minutes, continuous data on feeding behavior, and ad libitum data on social interactions (Wilson 2012). Additionally, in the larger Kasekela community, follows on family groups (mother, infant, and next oldest offspring) have been conducted since 1970 with the aim of targeting each family once per month. During these follows, a team of two researchers record record 1-minute point samples on the mother, her youngest offspring, and (when possible) observable older siblings. These point samples indicate proximity between the mother, infant and sibling (see distance categories below) and on-the-minute occurrence of various behaviors (see Lonsdorf et al. (2014) for a detailed ethogram).

In addition to the focal behavioral data described above, we screened four other data sources that contained descriptive information about some of the cases:

1. Attendance records: these records were collected from 1965-2001 in Kasekela and 1985-2001 in Mitumba. Attendance records document each chimpanzee’s arrival and departure from a feeding station along with narrative notes of target events such as social interactions and health issues.
2. Goodall field journals: these were used primarily in the early years of the study (from 1960-1968) and contained narrative descriptions of events in the Kasekela community.
3. Character files: these are compilations of primary observations and secondary extractions from other data sources of events specific to an individual chimpanzee (compiled from 1964 to 1989).
4. Ad hoc field reports: these are reports of specific notable events, such as infanticide attacks, disease outbreaks, etc.

*Categorization of causes of death*

Table S1 provides a cause of death categorization adapted from previous work on mortality in this population (Williams et al. 2008, Wilson et al. 2014). It is important to note that a substantial numbers of infants simply disappear. For some of these, we assigned their death to certain categories based on specific evidence. For example, in the case of a mother returning without her infant and with injuries consistent with a chimpanzee attack, we assigned “Disappeared-infanticide”. If a mother reappeared without her infant after either she or the infant exhibited signs of illness, we assigned “Disappeared-illness”.

*Definitions of behavioral interactions with corpses*

1. Atypical transport – transporting of the corpse in a manner other than typical dorsal/ventral carrying; this includes transport in the mouth, hand, foot, neck pocket, groin pocket, over the shoulder, or dragging on the ground. Differs from normal transport of an infant, which is either clinging ventrally or riding dorsally (Goodall 1967).
2. Peer – close visual inspection without contact
3. Smell – olfactory investigation of the corpse without contact
4. Inspect (Body, Face, or Genitals/Anus) – direct contact with a particular body area, may include staring and smelling
5. Manipulate – non-aggressive movement of a body part (e.g. lifting and lowering a limb)
6. Groom – parting of the hair with hands fingers or lips, removal of debris or ectoparasites
7. Sexual – mounting of corpse and/or interactant rubbing its anogenital region with the corpse
8. Play – using the corpse as an object during play, including tossing, bouncing, using corpse to tickle oneself
9. Rough handling – includes displaying with, hitting, throwing, stepping on corpse (as in Stewart et al. 2012), does not include dragging, which is included in ‘atypical transport’
10. Swat flies – swift hand/arm movement directed at flies gathering around the corpse
11. Eat – ingesting portions of the corpse

RESULTS

*Causes of death by age*

Table S2 displays counts and percentages of causes of death by age class.

*Example narrative descriptions of interactions*

Investigatory behaviors with contact:

- *LB (sibling of BH) taps on its chest with her knuckles gently, about 5 times*
- *FO (sibling of FI) carries FI’s dead body and travels 6 meters, then sit down and grooms the dead body, hit/pet several times using fingers on FI belly.*

Play:

- *GK (sibling of GR) turns a sideways somersault ending so that her bottom is near corpse, lying on her back. Turns sideways to it and plays with its arm and leg. Pulls its hand into her neck, wiggles it there, and gives small grunt like laughs…(8 minutes later). Feels its tummy with both hands, flats of fingers. Picks up an arm as though to pull towards her, looking at Olly (mother). Lays it down. Puts her foot on it.*
- *TZN (unrelated to GAsb) throws the corpse up and down like a ball and hit it on a tree then climb tree with the corpse and continue to play with it*

Rough handling:

- *Fifi (unrelated to OLb1) is lying on her back in the nest and she is holding the dead chimp by one arm and letting it dangle over the edge of the nest. Then she holds the legs with her foot and shakes it back and forth and slaps at it with her hand.*

SUPPLEMENTARY REFERENCES

Goodall J. 1967 Mother-offspring relationships in chimpanzees. In: *Primate ethology* (ed D

Morris), pp. 287–345. London: Weidenfeld & Nicolson.

Lonsdorf EV, Markham AC, Heintz MR, Anderson KE, Ciuk DJ, Goodall J, Murray CM. 2014

Sex differences in wild chimpanzee behavior emerge during infancy. *PLoS ONE*, 9, e99099. (https://doi.org/10.1371/journal.pone.0099099)

Stewart FA, Piel AK, O’Malley RC. 2012 Responses of chimpanzees to a recently dead

community member at Gombe National Park, Tanzania. *Am. J. Primatol.***74**,1-7.

Williams JM, Lonsdorf EV, Wilson ML, Schumacher‐Stankey J, Goodall J, Pusey AE. Causes of

death in the Kasekela chimpanzees of Gombe National Park, Tanzania. *Am. J. Primatol.* **70**, 766–77. (<http://dx.doi.org/10.1002/ajp.20573>)

Wilson ML. 2012 Long-term studies of the chimpanzees of Gombe National Park, Tanzania.

In *Long-term field studies of primates* (eds P Kappeler, D Watts), pp. 357-384. Berlin: Springer.

Wilson ML, Boesch C, Fruth B, Furuichi T, Gilby IC, Hashimoto C, et al. 2014 Lethal

aggression in Pan is better explained by adaptive strategies than human impacts. *Nature* **513**, 414–417. (http://dx.doi.org/10.1038/nature13727).
