# Supplementary material for: Why chimpanzees carry dead infants: an empirical assessment of existing hypotheses
Source: R Soc Open Sci. 2020 Jul 1;7(7):200931. doi: 10.1098/rsos.200931 (PMC7428235; doi:10.1098/rsos.200931)
Supplement: Supplementary Tables [file rsos200931supp2.docx]

| Cause of death | Definition | Cause of death - attributed |
| --- | --- | --- |
| Disappeared | Infant disappears with no evidence of illness, injury or infanticide | Disappeared |
| Disappeared – infanticide | Infant disappears and mother returns with wounds suggestive of an attacked (following Wilson et al. 2014 ‘suspected’) | Infanticide |
| Disappeared – illness | Infant noted as unwell before disappearance or mother reappeared without infant in poor condition herself | Poor health |
| Disappeared with mother | Infant and mother both disappear | Disappeared with mother |
| Disappeared with mother - outbreak | Infant and mother both disappear during an outbreak | Poor health |
| Infanticide | A fatal attack by chimpanzees was either observed or inferred from strong evidence (following Wilson et al. 2014 ‘observed’ or ‘inferred’). | Infanticide |
| Illness | Infant was observed with ill health symptoms (diarrhea, respiratory, mange) prior to death (following Goodall 1983, Williams et al. 2008) | Poor health |
| Inadequate maternal care – other | Infant was taken from the birth mother by another chimpanzee and did not survive | Inadequate maternal care |
| Inadequate maternal care – own | Infant’s own mother provide inadequate care | Inadequate maternal care |
| Orphan | Mother predeceased infant | Orphan |
| Never seen alive | Infant was dead when first observed (unknown whether infant was born alive) | Never seen alive |
| Stillborn | Infant known to be stillborn from necropsy evidence | Never seen alive |
| Unknown | A corpse was observed but cause of death could not be determined | Unknown |
| Injury | Infant observed with wounds not consistent with a chimpanzee attack prior to death | Poor health |
| Poor health | Infant was indicating distress prior to death (excessive crying) but no observed cause could be determined or infant died during a period when the mother was observed to be ill. | Poor health |
| Human killing | Infant corpse found with evidence of human killing | Human killing |

Supplementary Table 1. Causes of death and definitions. For analyses of duration of carrying, causes of death were combined into five categories of “Causes of death-attributed”: infanticide, poor health, inadequate maternal care, unknown, never seen alive. Note that individuals in any “Disappeared” categories were excluded from analyses due to the absence of a corpse.

Supplementary Table 2. Causes of death by age class for all categories.

| Corresponding Hypothesis | COD (Infanticide) | COD (Never seen alive) | COD (Poor health) | COD (Unknown) |
| --- | --- | --- | --- | --- |
| Unawareness (COD) | -0.457 | -0.561 | -0.680 | -0.634 |
| Unawareness (COD) + Unawareness (maternal experience) | -0.936 | -1.01 | -1.06 | -0.199 |
| Unawareness (COD) + Slow  decomposition | -0.121 | -0.343 | -0.190 | -0.692 |
| Unawareness (COD) + Post-parturient condition/length of bonding | -0.465 | -0.565 | -0.669 | -0.603 |
| MAP | -0.000351 | -0.000434 | -0.000483 | -0.000404 |
| 2.5% | -2.29 | -1.81 | -2.11 | -3.20 |
| 97.5% | 1.31 | 0.601 | 0.755 | 2.06 |

Table S3. Estimates for individual Cause of Death (COD) categories from the model comparison. The reference group is COD

(inadequate maternal care). Model-averaged parameter estimates (MAP) with upper (97.5%) and lower (2.5%) bounds of the 95% confidence intervals are given in the bottom rows.
